# Design of non-pharmaceutical intervention strategies for pandemic influenza outbreaks

**DOI:** 10.1186/1471-2458-14-1328

**Published:** 2014-12-29

**Authors:** Dayna L Martinez, Tapas K Das

**Affiliations:** Department of Mechanical and Industrial Engineering, Northeastern University, 360 Huntington Avenue, Boston, MA USA 02115; Industrial and Management Systems Engineering, University of South Florida, 4202 East Fowler Avenue, ENB 118, Tampa, FL USA 33620

**Keywords:** Pandemic influenza, Mitigation strategies, Non-pharmaceutical interventions

## Abstract

**Background:**

As seen during past pandemic influenza outbreaks, pharmaceutical interventions (PHIs) with vaccines and antivirals are the most effective methods of mitigation. However, availability of PHIs is unlikely to be adequate during the early stages of a pandemic. Hence, for early mitigation and possible containment, non-pharmaceutical interventions (NPIs) offer a viable alternative. Also, NPIs may be the only available interventions for most underdeveloped countries. In this paper we present a comprehensive methodology for design of effective NPI strategies.

**Methods:**

We develop a statistical ANOVA-based design approach that uses a detailed agent-based simulation as an underlying model. The design approach obtains the marginal effect of the characteristic parameters of NPIs, social behavior, and their interactions on various pandemic outcome measures including total number of contacts, infections, and deaths. We use the marginal effects to establish regression equations for the outcome measures, which are optimized to obtain NPI strategies. Efficacy of the NPI strategies designed using our methodology is demonstrated using simulated pandemic influenza outbreaks with different levels of virus transmissibility.

**Results:**

Our methodology was able to design effective NPI strategies, which were able to contain outbreaks by reducing infection attack rates (IAR) to below 10*%* in low and medium virus transmissibility scenarios with 33*%* and 50*%* IAR, respectively. The level of reduction in the high transmissibility scenario (with 65*%* IAR) was also significant. As noted in the published literature, we also found school closure to be the single most effective intervention among all NPIs.

**Conclusions:**

If harnessed effectively, NPIs offer a significant potential for mitigation of pandemic influenza outbreaks. The methodology presented here fills a gap in the literature, which, though replete with models on NPI strategy evaluation, lacks a treatise on optimal strategy design.

**Electronic supplementary material:**

The online version of this article (doi:10.1186/1471-2458-14-1328) contains supplementary material, which is available to authorized users.

## Background

Influenza pandemics have occurred on average three times in each century since the 1500s. There is an ominous expectation that a severe pandemic could occur and infect between 20 to 47 million people in the U.S. alone. In the absence of any control measures, it was estimated that it could cause around 200,000 deaths, 700,000 hospitalizations, 42 million outpatient visits, and an economic impact ranging between $71.3 and $166.5 billion in the U.S. [1]. More recent economic loss estimates are likely to be much higher. A potent influenza pandemic emergency crisis would likely last much longer than most other emergency events, and the resources needed to address the crisis such as supplies of vaccines, antiviral drugs, healthcare providers, hospital beds and medical supplies would be limited. Hence, strategic pandemic preparedness is a major concern, as we must be reasonably assured that we can contain a pandemic influenza outbreak. Currently there are many influenza viruses circulating in different parts of the world with the potential to mutate into highly pathogenic forms for which there is no immunity in the current human population. The most notorious ones being the avian influenza or bird flu, H5N1 and H7N9. WHO has reported 650 confirmed cases of H5N1 infection since 2003, of which 386 have died (per recent report of January 2014). There have also been recent reports of human infection with A(H7N9) since May 2013. WHO has reported in February of 2014 a total of 112 cases of H7N9 including 43 deaths.

In scientific literature, pandemic containment is defined as keeping the number of new infections under control by lowering the reproduction number under one (*R*_0_<1) or reducing the infection attack rate (IAR) under 10% (*IAR*<0.1). Known approaches for pandemic influenza and mitigation containment utilize both pharmaceutical interventions (PHIs) and non-pharmaceutical interventions (NPIs). PHIs include vaccines and antiviral drugs. NPIs include social distancing, quarantine, isolation, school and workplace closure, and travel restrictions.

As seen during the past influenza pandemics, the most effective mitigation measure is vaccination. However, the use of vaccination at the early critical stages of an influenza pandemic has major challenges due to our inability to accurately predict the nature of the virus strain. Other limitations include a timely development of a vaccine, availability and deployment of surge production capacity, and distribution strategy and logistics. For example, during the 2009 H1N1 outbreak, the development, production, and distribution of a vaccine took nine months [2, 3].

Antivirals can also offer an effective treatment and containment measure. However, it would require a substantial level of stockpile for an effective antiviral prophylaxis campaign. Such a campaign may be infeasible due to its prohibitive production and storage costs [4–7]. Moreover, the use of a large-scale antiviral-based prophylaxis strategy can result in some virus strains becoming antiviral resistant while maintaining infectiousness [8–10]. This could pose a major threat since, at present, antivirals are the only means for treating influenza.

NPIs, on the other hand, though often with some delays, have the advantage of being available at the early stages of a pandemic outbreak. Application of NPIs, and the resulting impact in limiting the early spread of the virus, can ease pressure on the health services providers while they develop, procure, distribute, and administer vaccines and antivirals [11]. NPIs are also likely to be the only effective containment measures in underdeveloped countries that may lack adequate resources for reasonable vaccination and antiviral campaigns [12].

Some of the NPIs (e.g., social distancing) are already incorporated by many countries in their national pandemic preparedness plans [13–17]. Other major organizations that have also included NPIs in their preparedness plans and guidelines are the World Health Organization (WHO) [18] and the Centers for Disease Control and Prevention (CDC) [16]. However, our review of the above plans and guidelines reveals that these vary significantly in when and how to implement the NPIs. The variations can be seen both in their basic definitions as well as recommendations for declaration thresholds, implementation stages, target population, and implementation logistics.

Some of the recent papers have used agent-based (AB) simulation models for pandemic influenza to examine the efficacy of non-pharmaceutical intervention strategies. A review of these papers reveals that there exists significant variabilities in the assumptions made in these studies regarding some of the key model parameters, such as intervention initiation, duration of the intervention phases, composition of risk groups, compliance levels, and other NPI related parameters (e.g., partial/full school closure, community contact rate increase during school closure [19]). As a consequence, the reported usefulness of the NPIs also vary significantly.

Mathematical models have also been employed to analyze effectiveness of the NPIs [20–25]. However, mathematical approaches are not well adapted to modeling aspects like detailed demographic and geographic features, daily schedules of people, the process of individual to individual transmission, and tracking infection spread. Hence, mathematical models can only obtain aggregate estimates of basic reproduction number (*R*_0_) and infection attack rates (IAR). AB simulation models, on the other hand, can consider demographic and geographic features of the region, individual health and family status, and daily schedules. AB models also account for infection spread resulting from individual interactions using a detailed infection-transmission model, and thus yield better estimates of *R*_0_ and IAR.

In what follows, we first give a brief outline of an AB model. We then discuss/present some of the defining components of the AB model concerning virus epidemiology, social behavior, and non-pharmaceutical interventions. Thereafter, we discuss the NPI strategy design approach. We demonstrate the efficacy of the design approach on simulated outbreaks of pandemic influenza with different levels of virus transmissibility.

## Methods

Our methodology uses an AB simulation model, an earlier version of which was presented in Uribe et al. [26]. The AB simulation model tracks each individual in the outbreak region and their daily activity schedules. In addition, the AB model considers a variety of mixing groups, a detailed contact and infection transmission model, disease natural history, and a number of mitigation and containment actions.

### Agent based simulation model

The AB model begins by creating mixing groups and individuals. Individuals are created with a set of attributes based on demographic data. Adults and children have the following common attributes: age, gender, household, health condition (poor, moderate, good), and disease status (infected or non-infected). Other attributes for adults include parenthood and workplace, and for children its the school type.

Mixing groups include households (characterized by the number of adults and children), workplaces (offices, factories, stores, educational institutions, and restaurants), entertainment centers, and churches. Hourly activity schedules are assigned to each individual based on their attributes. These schedules differ between weekdays and weekend days.

As the AB simulation progresses through the hours of the day, the model traces the movement of every individual among the mixing groups and track their contacts. The pandemic influenza is triggered by introducing a limited number of infected cases into the region. Upon contact with an infected, a susceptible may become infected with a probability that is determined by the infection-transmission model (discussed later). The following are some of the defining components of the AB model.

#### Disease natural history

**Figure 1:**
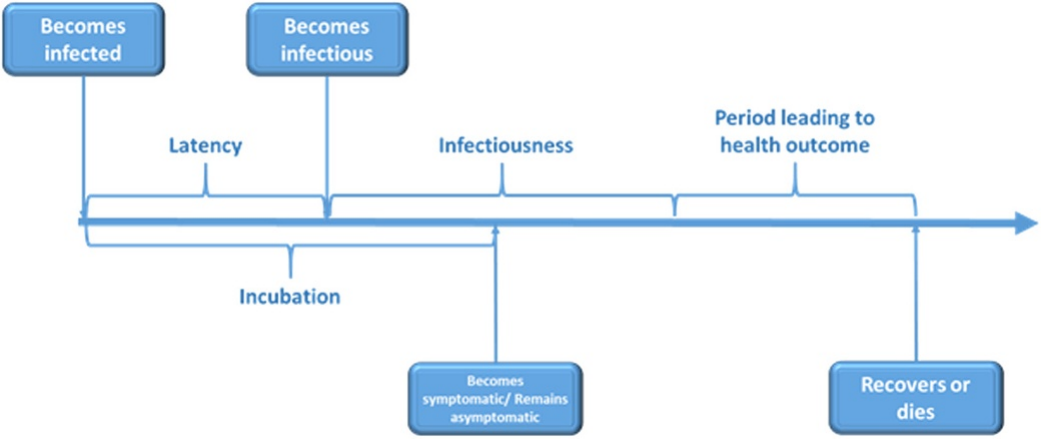
**Influenza disease natural history.** Typical influenza disease natural history showing the progression of the disease from the moment of exposure until health outcome.

The AB model considers a disease natural history as depicted in Figure 1. When a susceptible individual becomes infected, s/he enters the latency and incubation period simultaneously. Infectiousness starts at the end of the latency period and symptoms show at the end of the incubation period. It is considered that some infected individuals may remain asymptomatic. After the infectiousness period is over, an individual either recovers or dies with a certain probability. We assume that recovered individuals develop immunity and are not susceptible.

#### Contact process

The hourly schedule of each individual dictates which mixing group s/he will belong to at any given hour of a day. The members in each mixing group in any hour is comprised of a number of susceptible and possibly some infected individuals. These numbers determine the number of contacts, which is obtained using the contact probabilities given in Germann et al. [27]. Contact probabilities depend on age and the type of mixing group. For example, an infected child contacting a susceptible child in a household will have a different probability than an infected adult contacting a susceptible adult in a workplace. The infection transmission model, described next, determines how contacts result in infections.

#### Infection transmission model

**Figure 2:**
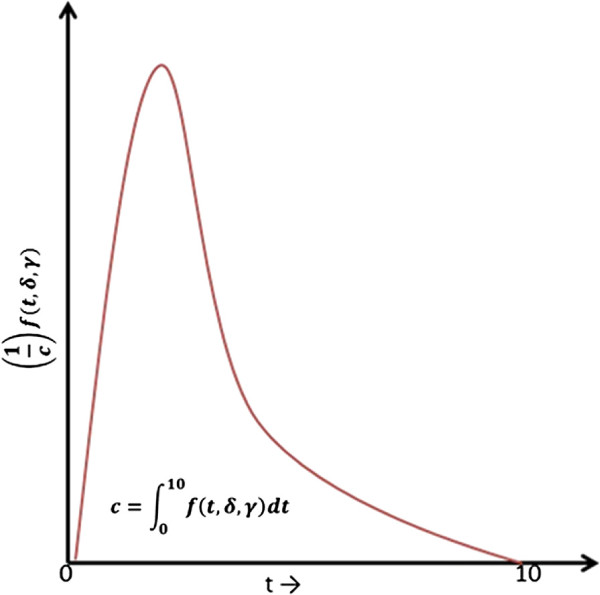
**Profile of infectiousness.** Typical time varying profile of influenza infectiousness.

When an individual *j* becomes infected, s/he enters into a latency period. At the end of the latency, the period of infectiousness begins. During this period, infectiousness first increases and then decreases, which is assumed to follow a lognormal distribution function [4]
1

where *t* denotes the elapsed time of the infectiousness period in hours, *δ* and *γ* are the distribution parameters. As shown in Figure 2, we use a truncated (at *t = 10* days) version of the lognormal distribution function based on the assumption that infectiousness does not last more than 10 days. Hence, the amount of virus shed by the *j*^*th*^ infected individual during a time interval of infectiousness is given by the area under the curve *f*(*t*,*δ*,*γ*) during the time interval multiplied by *ρ*_*k*_, a calibrated parameter that determines the virus transmissibility scenario (k = low, medium or high).

Let at any hour *t*, *t*_*j*_ denote the hour of infection of the *j*^*th*^ infected individual. The amount of virus that is ingested by a susceptible contact *i* from the *j*^*th*^ infected until hour *t*_*j*_+1 is given by . It is assumed that the amount of viral shed is divided equally among the total number of contacts *n*_*j*_(*t*) of the *j*^*th*^ infected at hour *t*. Then we have that
2

where +1 in the denominator indicates that the *j*^*th*^ infected individual will re-ingest a portion of the virus shed, and *c* denotes the truncation coefficient and given by .

A susceptible individual *i* may have contacts with a set  of infected individuals during any hour *t*, where each of the infected individuals is at a different day of their infectiousness period. During any contact period beginning at time *t* and ending at *t*+1 (which we refer to as hour *t*), the susceptible individual *i* will accumulate a viral load equal to the sum of the ingested virus from each one of its infected contacts. Then the total viral load of susceptible *i* accumulated during the hour *t* is given as
3

**Figure 3:**
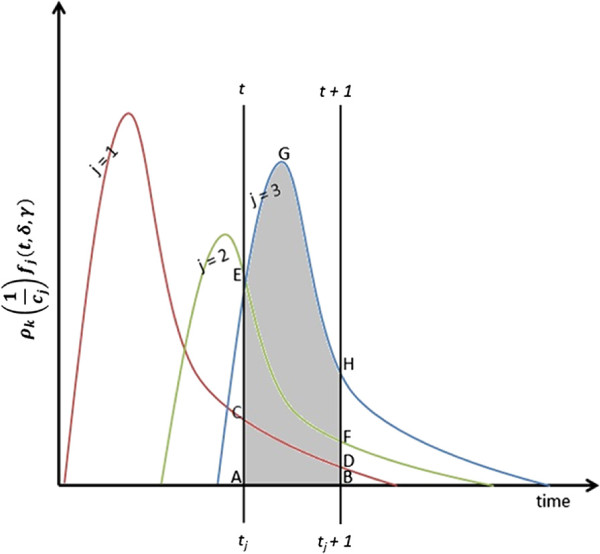
**Viral load accumulation.** Graphical representation of viral load accumulation by a contact from multiple infected in a mixing group.

Figure 3 presents a pictorial illustration of a susceptible individual *i* that has been contacted by three different infected (*j*=1,2,3) during a period of time starting at *t* and ending at *t*+1. Note that the *y* axis in the figure denotes a normalized value  such that the area under the curves represent the total virus shed by the infected individuals. The virus shedding profile distribution parameters *δ* and *γ* are also influenced by the virus epidemiology. At the time *t*, the elapsed period of infectiousness for three infected are *t*_1_, *t*_2_, and *t*_3_, respectively. Infected *j*=1 will shed a total amount of virus given by the area ACDB. Similarly, for infected *j*=2 and 3, the total amount of virus shed will be given by the areas AEFB and AEGHB, respectively. The sum of these three areas represents the total amount of virus shed by the three infected. The proportion of this total amount that will be ingested by the susceptible *i* depends on the number of other contacts of these three infected during the hour *t*.

We assume that a susceptible individual *i*, who does not get infected during hour *t*, keeps accumulating viral load through the hours of a day until either an infection is triggered or the day ends. Then, the total viral load accumulation for a susceptible *i* until hour *t* of the day is given by
4

We also assume that for a susceptible contact who is not infected by the end of the day, the value of the total viral load accumulation becomes zero at the start of the following day. To our knowledge, no method currently exists that would enable us to model the process of viral load depletion in real time inside a human body through immune response or inability of virus to enter a healthy cell.

Since any of the numerous virus particles ingested by a susceptible can trigger an infection, we model the infection process as a Poisson process with the total accumulated viral load as the rate of infection. Let *T*^*i*^ denote the random variable defined as the time required for a susceptible contact *i* to get infected. Then *T*^*i*^ is exponentially distributed with a rate of *λ*^*i*^(*t*)=*VLA*^*i*^(*t*).

Then, the probability that the susceptible *i* will get infected during the hour *t* is
5

where *α*<1 is an age based factor [4] and it gets closer to one for susceptible contacts of higher age, as well as young children.

### Non-pharmaceutical interventions (NPIs)

In our AB simulation model, we consider four different non-pharmaceutical intervention actions. These actions are case isolation, household quarantine, school closure, and workplace closure. In this section we discuss each one of these by providing their definitions, key implementation parameters, and how they are incorporated in the simulation.

*Case isolation* refers to household confinement of symptomatic individuals. The infected diagnosed by a doctor are expected to obey isolation, when in effect, with a certain compliance probability. The compliance probability is assumed to depend on the extent of illness and work status. Using expert opinion (from practicing physicians in Tampa, FL, U.S.A.) we divided the period of infectiousness in three phases. Phase 1 consists of the first 2 days with a 30% probability of being too ill to continue with regular schedule, phase 2 covers the next 3 days with a 80% probability, and phase 3 is the remaining 5 days with a 30% probability.

The probability of obeying isolation also depends on the work status of an infected [28]. An unemployed individual has a higher probability of obeying than an employed. Therefore, case isolation compliance probability is obtained as the product of the probability that the individual is too ill to continue with his/her regular schedule and the probability that the individual will obey the isolation recommendation.

If an individual complies with isolation, then it is assumed that s/he stays at home all day. If an individual does not comply with isolation then s/he follows regular schedule. If an individual is employed, does not comply with isolation, and his/her workplace is closed, then the individual is assumed to stay at home but spend five hours for errands out of home.

Children (below 18 years of age) are assumed to fully comply with isolation. A child younger than 13 is assumed to be supervised by an adult when isolated at home. If there is a stay at home adult, that adult takes care of the child. However, if there are no stay at home adults in the household, then the AB simulation randomly selects a working adult member of the household to provide supervision.

In the AB simulation model, we use three parameters to characterize case isolation: isolation threshold (time between disease diagnosis and beginning of isolation), isolation duration (dictated by the disease natural history), and isolation compliance (influenced by demographics).

*Household quarantine* is a measure to restrict movement of household members of an infected case. Note that the infected case is one who has been diagnosed by a doctor and who is in compliance with isolation. Household quarantine compliance probabilities for uninfected household members are adopted from [28], which depends on employment status. If a quarantined household have members that are 13 years or younger, they stay at home with a 100% probability under adult supervision.

If an uninfected member of a quarantined household complies with the measure, her/his schedule changes to stay at home without any errands. If an individual is non-compliant and his/her workplace or school (for those older than 13 years) is not closed, then that individual continues with the regular assigned schedule. If the workplace/school is closed, then s/he is assigned a new daily schedule for staying home with five hours of errands. We parameterized household quarantine in our AB simulation model using quarantine initiation threshold, duration, and compliance.

*School closure* is modeled using a partial school closure approach. We divide the school into smaller mixing groups consisting of individual classrooms. Children belonging to these smaller mixing groups (classrooms) are considered to remain in it all the time except during the lunch hour when they interact with other classroom children. A school closes when one or more classrooms are closed. A classroom is closed when a threshold of new infected in the classroom is reached. When a school is closed, students fully comply with closure and stay at home. Students younger than 13 stay at home with an adult.

We implemented the above school closure approach using three key parameters: number of infected cases to close a class, number of closed classes to close a school, and closure duration. Note that a class/school opened after its closure duration could close again if the above thresholds are met by new infections.

*Workplace closure* is modeled after school closure, where a workplace and its various departments are treated as a school and classrooms, respectively. If an individual’s workplace is closed, and s/he is neither subjected to isolation or household quarantine nor supervising a child as discussed earlier, then that individual follows the stay at home schedule with five hours of errands. However, if the individual is subject to isolation or household quarantine, then the rules of these interventions apply. The partial closure strategy for schools and workplaces has not been considered in the published literature.

### NPI strategy design approach

We adopted a statistical approach for the design of optimal NPI strategies. We used a highly fractional factorial experiment design with sixteen (16) NPI parameters as factors and a number of performance measures including total number of infected, total number of deaths, total number of contacts, and total cost. The experiments were conducted using our AB simulation model. We first performed screening experiments with all factors at 2 levels. Factors found to be significant in the screening experiments were further examined with 3-level experiments. Using the results of the fractional factorial experiments, we developed regression equations linking the NPI parameters to various performance measures. These regression equations were then optimized to obtain NPI strategies.

Table 1 presents a summary of all the factors, their acronyms, and the units of measurement. Two of the factors in Table 1 that were not described earlier are as follows. *Global threshold* is the number of cases needed for public health officials to declare an outbreak of pandemic influenza in a region requiring deployment of interventions. *Deployment delay* is the time needed for non-pharmaceutical intervention measures to be fully deployed once a pandemic outbreak is declared.

**Table 1:** **Factors and their values considered in the 2** ^**16-7**^ **factorial experiments**

Factor	Acronym	Measurement unit	Low level	High level	References
Global threshold	GT	Integer	10	50	[6, 29–31]
Deployment delay	DD	Days	3	7	
Case isolation threshold	CIT	Days	0	1	[4, 11, 19, 27, 29, 32, 33]
Case isolation duration	CID	Days	7	10	[4, 11, 19, 27, 29, 32, 33]
Case isolation compliance					
for workers	CICW	Percentage	53	75	[4, 19, 29, 33]
Case isolation compliance					
for non-workers	CICNW	Percentage	57	84	[4, 19, 29, 33]
Household quarantine					
threshold	HQT	Days	0	1	[4–6, 27, 29]
Household quarantine					
duration	HQD	Days	7	10	[4–6, 27, 29]
Household quarantine					
compliance for workers	HCW	Percentage	53	75	[4–6, 29]
Household quarantine					
compliance for non-workers	HCNW	Percentage	57	84	[4–6, 29]
*#* of cases to close a					
class in a school	CCC	Integer	1	3	[2, 4, 27, 31–34]
*#* of classes to close					
a school	CCS	Integer	1	3	
School closure duration	SCD	Days	21	42	[2, 19, 27, 29, 31–36]
*#* of cases to close a					
department in a workplace	CCDW	Integer	3	5	[2, 4, 32, 33, 35]
*%* departments to close					
a workplace	PDCW	Percentage	30	50	
Workplace closure duration	WCD	Days	7	14	[2, 19, 32, 33, 35]

Table 1 also presents the references for high and low levels of all NPI factors. Deployment delay (DD), number of classes to close a school (CCS), and percentage of departments to close workplaces (PDCW) don’t have any references cited. We decided to examine DD as a factor encouraged by the fact that during the H1N1 2009 pandemic, even though school closure was deployed after a threshold of infected cases was reached, there was a delay until it was fully implemented. Consideration of CCS and PDCW is new as the partial closures of schools and workplaces have not been considered in the published literature.

## Results and discussion

### Implementation of NPI strategy design approach

We implemented our design approach on three pandemic influenza transmissibility scenarios: low (33% IAR), medium (50% IAR), and high (65% IAR). We considered the outbreak region to be the Hillsborough County of Florida, USA, with population of approximately 1.1 million.

### Two-level fractional factorial experiment

We used a 2^16−7^ factorial design with a total of 512 experiments in which all 16 main factors and all 2-level interactions were not confounded. We used the results of this experiment to screen the initial set of factors and select the significant ones to be included in the subsequent 3-level experiment. We ran 5 replicates (with different sets of seeds for random variables) for each of the experiments. Each simulation run took on average 15 minutes on a Dell quad core desktop computer with 8 GB RAM. Table 1 shows all factors and their values for low and high levels.

#### Low transmissibility scenario (33% IAR)

Table 2 presents the effects of the significant main factors and the 2-level interactions on the total number of infected as the measure of performance. As evident from the effects, school closure is the most significant non-pharmaceutical intervention. An increase from one to three in number of cases to close a class (CCC) resulted in a large increase (+8.04%) in the total number of infected. Doubling the school closure duration (21 days to 42 days) resulted in a large decrease (-3.75%) in the total number of infected. Since a majority of contacts and infections happens in schools among children, the above results were expected, which also support the results previously reported in the literature [4, 11, 27]. An outcome that was not anticipated is concerning the behavior of the case isolation threshold (CIT). Allowing individual cases to be isolated at home a full day after becoming symptomatic (instead of immediate isolation) actually resulted in a decrease in the total number of infected, instead of an increase. It was observed that immediate isolation at home of infected cases significantly increased the number of infections within the household. Consequently, it is more effective to allow an infected case on the first day of infectiousness to maintain a normal schedule of work and/or errands in the community, where the contact probabilities are much lower than at home.

**Table 2:** **Effects of the significant main factors and 2-level interactions on the total number of infected for the low transmissibility scenario**

Factors		Effects (on total # infected)	
**Main Factors**		**Change in total # infected when factor**	
		**changes from a low to a high level**	
DD		+0.57*%*	
CIT		−1.44*%*	
CCC		+8.04*%*	
CCS		+1.90*%*	
SCD		−3.75*%*	
CCDW		+1.28*%*	
WCD		−0.41*%*	
**Interactions**		**Effect of change in Factor 1 when Factor 2 is in**	
**Factor 1**	**Factor 2**	**Low Level**	**High Level**
CIT	CCC	−0.31*%*	−2.56*%*
CIT	SCD	−1.96*%*	−0.91*%*
CIT	CCDW	−1.05*%*	−1.82*%*
CCC	CCS	+7.36*%*	+8.73*%*
CCC	SCD	+10.81*%*	+5.27*%*
CCDW	CCS	+7.19*%*	+8.90*%*
CCS	SCD	+0.95*%*	+2.86*%*
SCD	CCDW	−2.55*%*	−4.94*%*

Legend: DD- deployment delay; CIT - case isolation threshold; CCC - number of cases to close a class in a school; CCS - number of classes to close a school; SCD - school closure duration; CCDW - number of cases to close a department in a workplace; WCD - workplace closure duration.

**Figure 4:**
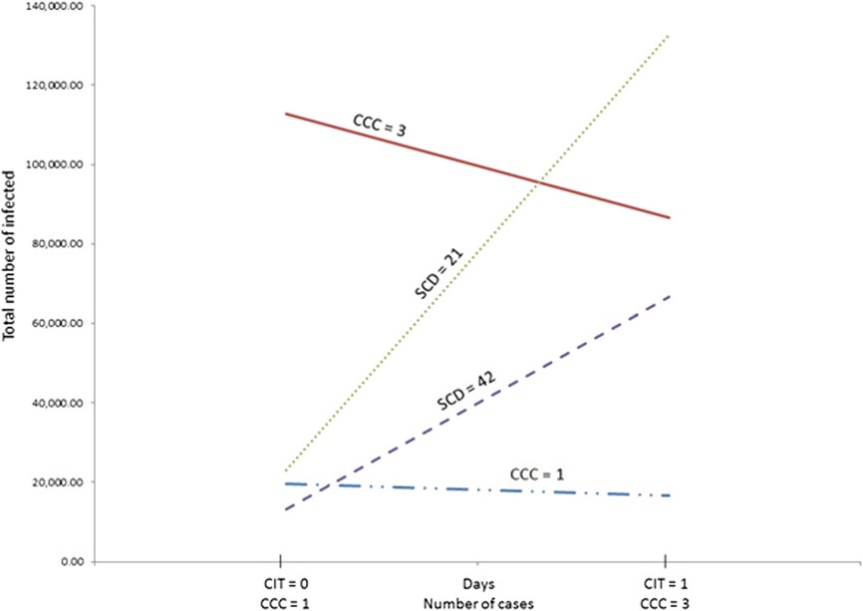
**Interaction effects from the 2-level experiment for the low transmissibility scenario.** Graphical representation of the interaction effects on the total number of infected for CCCxCIT and SCD x CCC obtained from the 2-level experiment for the low transmissibility scenario (CCC - cases to cose a classroom; SCD - school closure duration).

Significant interactions given in Table 2 can be interpreted as follows. For the interaction CIT x CCC, when CCC is kept in its low level (one), a change in CIT from its low level (0 day) to high level (1 day) results in a decrease in the total number of infected by -0.31%. Whereas, when CCC is in its high level (three), the corresponding decrease in the total number of infected is -2.56%. This represents a more than 8 fold increase in the reduction in the total infected when CCC is high (three). It is commensurate with the fact that high level of CCC causes 8.04% increase in the number of infections. The other most notable interactions are CCC x SCD and CCS x SCD. As shown in Figure 4, case isolation threshold (CIT) has a major impact on the total number of infections when the number of cases to close a classroom (CCC) in a school is 3 instead of 1. Also, impact of a higher CCC (=3) on the total number of infected is much lower when the closure duration (SCD) is higher (=42). Hence, if shorter school closure duration is desired, then classes should be closed quicker after an infection is discovered. Another interesting observation to note from interaction CCS x SCD (in Table 2) is that if a longer school closure duration is selected, then it is preferable to close a school sooner after a class is closed.

After identifying the significant main factors and interactions in the 2-level experiment, with the low transmissibility scenario, we developed a regression model for the total number of infected. The model has a low *R*^2^ value of 0.652. Even after including all other factors and interactions, the *R*^2^ value rose slightly to 0.6781. Low *R*^2^ value indicates that a linear regression over the NPI parameters does not sufficiently characterize the variations in the total number of infected. However, an optimization of the regression equation resulted in an NPI strategy (in Table 3) that significantly improved values of the response measure when compared to a baseline strategy (with no interventions), and an ad hoc NPI strategy. A detailed comparison of the optimal strategy (NPI ^∗^) with the baseline and ad hoc (NPI) strategies is presented in Table 4.

**Table 3:** **NPI** ^**∗**^ **strategy to minimize the total number of infected cases for the low transmissibility scenario (as obtained from the two-level fractional factorial experiment based design approach)**

Factor	Optimal	Factor	Optimal	Factor	Optimal
	value		value		value
GT	10	DD	7	CIT	1
CID	10	CICW	0.75	CICNW	0.57
HQT	1	HQD	7	HCW	0.53
HCNW	0.84	CCC	1	CCS	3
SCD	21	CCDW	3	PDCW	0.3
WCD	7				

Legend: GT - global threshold; DD - deployment delay; CIT - case isolation threshold; CID - case isolation duration; CICW - case isolation compliance for workers; CICNW - case isolation compliance for non-workers; HQT - household quarantine threshold; HQD - household quarantine duration; HCW - household quarantine compliance for workers; HCNW - household quarantine compliance for non-workers; CCC - number of cases to close a class in a school; CCS - number of classes to close a school; SCD - school closure duration; CCDW - number of cases to close a department in a workplace; PDWC - percentage departments to close a workplace; WCD - workplace closure duration.

**Table 4:** **Comparison of performance measures among the NPI** ^**∗**^ **, the baseline, and ad hoc NPI strategy using results from the 2-level experiment with the low transmissibility scenario**

Performance measure	Baseline	NPI	NPI ^∗^	Performance measure	Baseline	NPI	NPI*
IAR	33.06%	20.62%	1.83%	Infections 0-19 yrs.	225,467	156,849	14,345
CFR	0.69%	0.37%	0.03%	Infections 20-64 yrs.	91,959	43,135	3,346
Pandemic Duration (Days)	135	350	75	Infections 65-99 yrs.	17,645	8,975	857
Total Contacts	1,177,393	738,716	71,771	Infections Households	37,562	65,107	7,470
Contacts 0-19 yrs.	818,912	618,661	62,920	Infect. MG Types(1-2)	46,600	7,019	535
Contacts 20-64 yrs.	294,046	102,973	7,160	Infect. Schools	249,304	136,043	10,458
Contacts 65-99 yrs.	64,435	17,082	1,691	Infect. MG Types(9-12)	1,605	790	85
Contacts Households	238,684	344,169	40,213	Total Deaths	7,009	3,764	303
Contacts MG Types(1-2)	231,051	37,785	3,185	Deaths 0-19 yrs.	1,041	744	67
Contacts Schools	699,427	352,987	28,013	Deaths 20-64 yrs.	4,095	2,059	156
Contacts MG Types(9-12)	8,231	3,775	360	Deaths 65-99 yrs.	1,873	961	80
Total Infections	335,071	208,959	18,548				

NPI ^∗^ strategy is optimized for the total number of infected.

Legend: IAR - infection attack rate; CFR - case fatality ratio; MG - mixing group.

**Figure 5:**
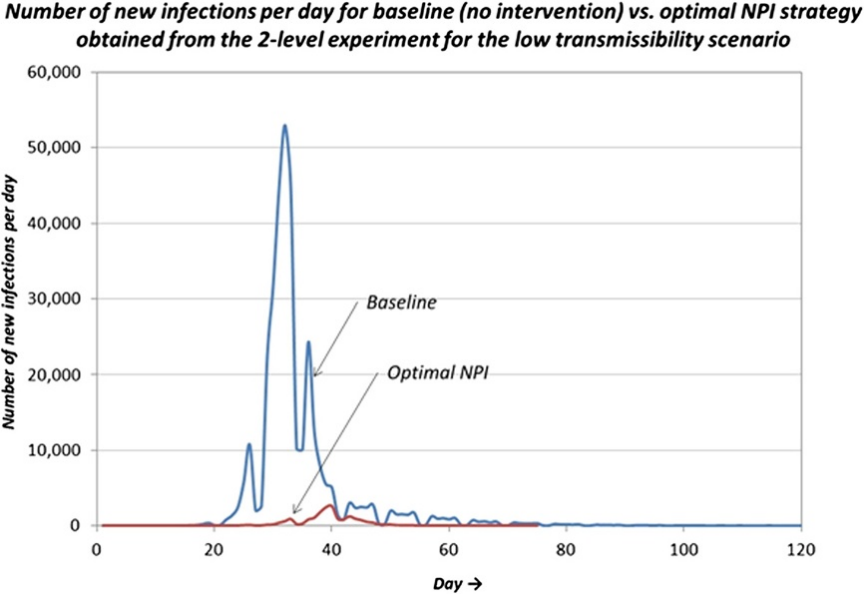
**Number of new infections per influenza pandemic day.**

Containment is achieved by the NPI ^∗^ strategy as the IAR is lowered from 33.06% (for baseline) to 1.83%. The NPI ^∗^ strategy also offered significant reductions in the CFR (case fatality ratio), the number of contacts, deaths, and infections in all age categories. Figure 5 depicts the total number of new infections per day during the length of the pandemic duration with low transmissibility. As evident from the figure, the NPI strategy not only reduced the total number of infections, it also significantly reduced the peak of infections as well as the length of pandemic duration.

#### Medium transmissibility scenario (50% IAR)

An analysis of variance driven design approach identical to that presented above for the low transmissibility scenario was repeated for an outbreak with a virus of medium transmissibility (50% IAR). The results for this scenario are presented in the Additional file 1: Tables S1, S2 and S3.

As in the low transmissibility scenario, school closure is the most significant intervention (see Additional file 1: Table S1). An increase in the number of cases to close a class (CCC) from one to three, resulted in an increase of 15.42*%* in the total number of infected. Also, an increase in school closure duration (SCD) from 21 days to 42 days decreased the total number of infected by -5.56%. Workplace closure factors were also found to be significant. However, their impacts on the total number of infected are not as notable as with factors related to school closure.

Significant interactions (Additional file 1: Table S1), included the one between global threshold (GT) and deployment delay (DD). This interaction shows the importance of surveillance (which impacts GT) and preparedness (which impacts DD). When interventions are not ready to be deployed promptly after a pandemic declaration (i.e., DD is high), then the necessary surveillance must be in place to accurately enforce GT and avoid delay in pandemic declaration.

The regression model for the total number of infected in the medium transmissibility scenario had an *R*^2^ value of 0.9144, which is a marked improvement over the low transmissibility scenario. That is, in the medium transmissibility scenario, the non-pharmaceutical interventions have a much more significant impact on the total number of infected. The optimal NPI strategy (NPI ^∗^) is shown in Additional file 1: Table S2. The impact of the NPI ^∗^ strategy, as compared to the baseline strategy and an ad-hoc NPI strategy, on different performance measures, is shown in Additional file 1: Table S3. The NPI ^∗^ strategy contains the pandemic by reducing IAR to 3.42*%*. Additional file 2: Figure S1 depicts how the NPI ^∗^ strategy reduces the total number of infections and the peak of new infections. It also shows that under NPI ^∗^, the outbreak experiences multiple recurrences of new infections and thus a longer pandemic duration. The recurrences were found to coincide with the re-openings of schools and workplaces.

#### High transmissibility scenario (65% IAR)

The results from this scenario are presented in Additional file 1: Tables S4, S5 and S6. As in other scenarios, school closure is the most significant intervention approach. As with the medium transmissibility scenario, the results for the high transmissibility scenario also emphasize the importance of the dependency between surveillance and preparedness. The regression model for this scenario has the highest *R*^2^ value of 0.9508. However, the optimized NPI (NPI ^∗^) strategy though effective in reducing infections, deaths, and contacts (see Additional file 1: Table S6) failed to contain the pandemic. Note that the IAR was reduced to only 16.97*%*. A pandemic is considered to be contained when IAR falls below 10%. It was also noted (see Additional file 3: Figure S2) that for the high transmissibility scenario, the NPI ^∗^ strategy greatly extends the pandemic duration with many recurrences. In such scenarios, a combination of NPIs with PHIs may provide the best approach for containment.

### Three-level fractional factorial experiment based design approach

**Figure 6:**
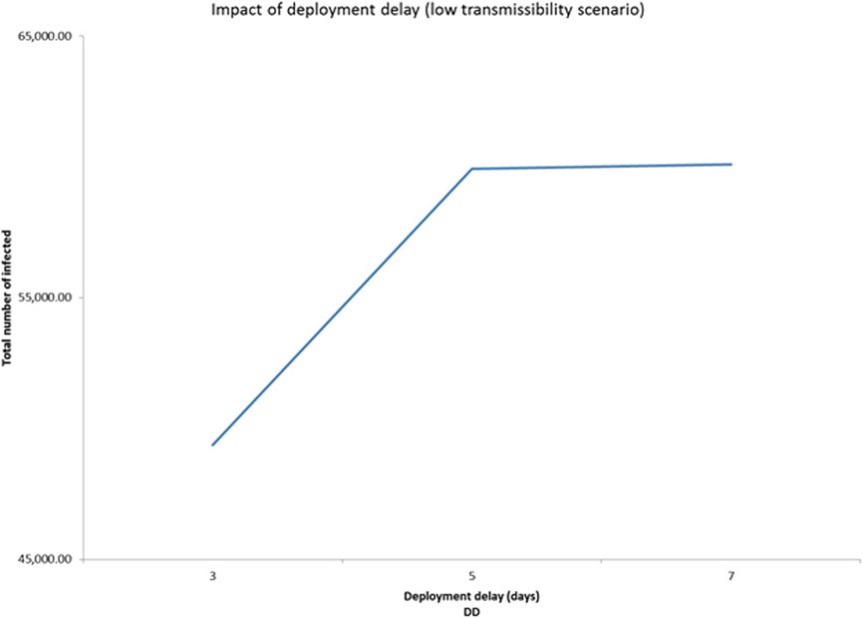
**Main factor effect for deployment delay (DD).** Impact of deployment delay in low transmissibility scenario (3-level experiment).

**Figure 7:**
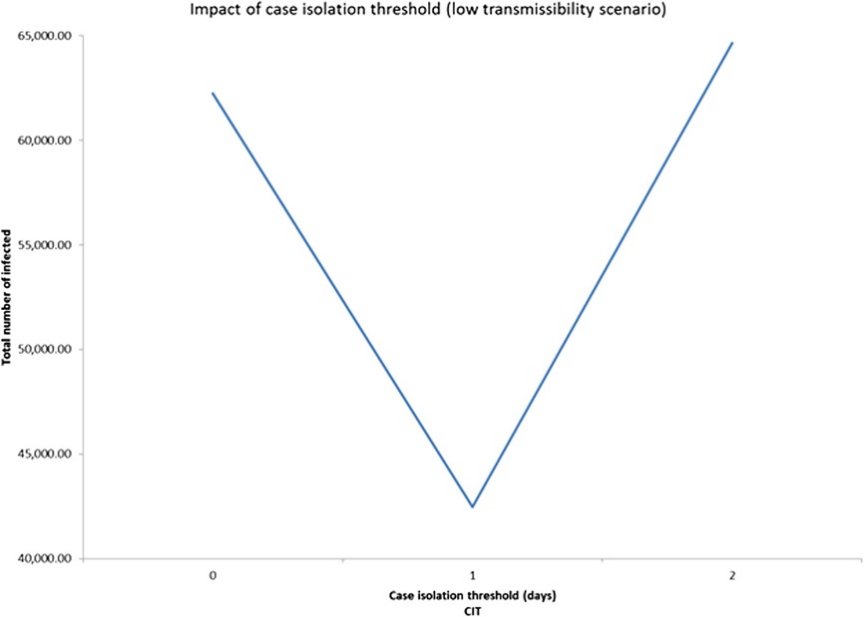
**Main factor effect for case isolation threshold (CIT).** Impact of case isolation threshold in low transmissibility scenario (3-level experiment).

**Figure 8:**
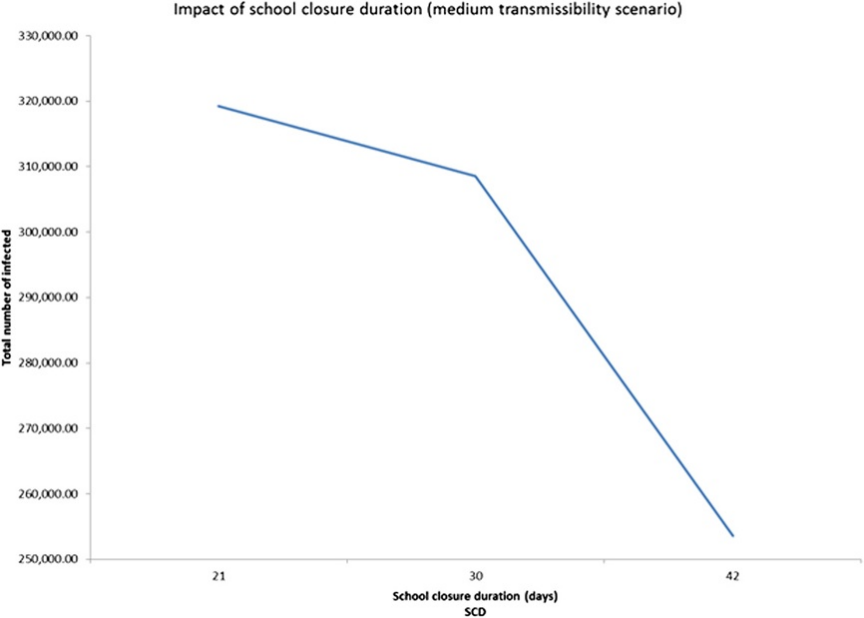
**Main factor effect for school closure duration (SCD).** Impact of school closure duration in medium transmissibility scenario (3-level experiment).

**Figure 9:**
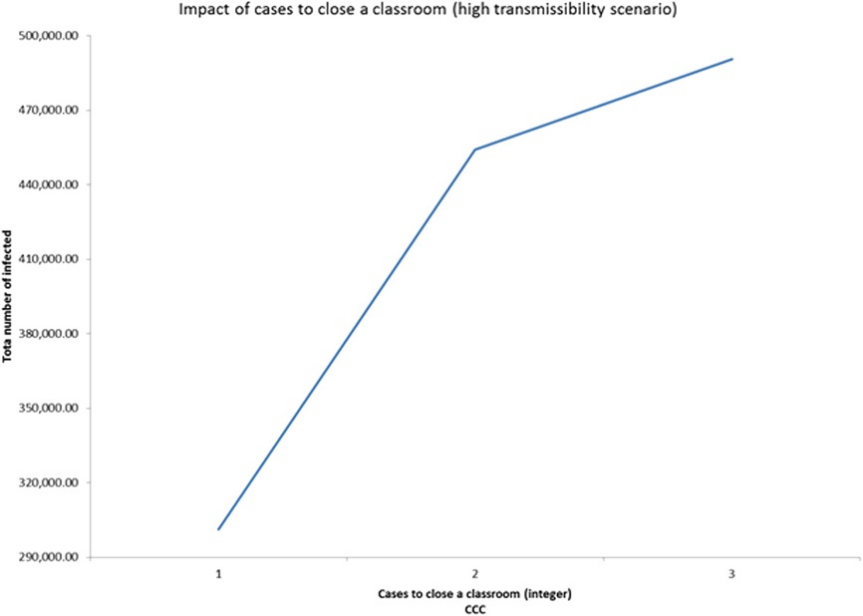
**Main factor effect for cases to close a classroom (CCC).** Impact of cases to close a classroom in high transmissibility scenario (3-level experiment).

In order to examine the presence of any non-linearity in the behavior of the significant main factors and interactions, we conducted 3-level fractional factorial experiments. The three scenarios have the following common factors: GT, DD, CIT, CCC, CCS, SCD, CCDW, and WCD. Additional file 1: Table S7 presents the values of the 3-levels for the above factors. Though the design can estimate effects of all main factors, it isn’t capable of estimating all the two-way interactions due to confounding. Though the results obtained from the 3-level experiment based design approach for each of the three transmissibility scenarios are similar to those obtained from the 2-level ANOVA approach, some interesting nonlinearities were observed as follows.It was observed from the low transmissibility scenario that increasing deployment delay from 5 (level 2) to 7 (level 3) days did not have a noticeable impact on the total number of infected (Figure 6). Adding a third level to case isolation threshold helped us to discover that while the number of infected decreases sharply as the threshold CIT increased from 0 to 1 day, the trend quickly reversed (see Figure 7) when the threshold was increased to 2 days.For the medium transmissibility scenario it was observed that school closure duration beyond 30 days produced a much sharper decrease in the total number of infected (see Figure 8). In the high transmissibility scenario, it was observed that a change from 1 to 2 for cases to close a classroom (CCC) has a very high impact on increasing the number of infected. However, a further increase of CCC to 3 has a much smaller impact on the rate of increase (see Figure 9).

## Conclusions

In this paper we have modeled pandemic influenza outbreaks using an agent-based simulation approach. The AB model incorporates detailed population demographics, dynamics of hourly schedules of people, a variety of mixing groups and their contact processes, infection transmission process, and a number of non-pharmaceutical interventions. Using a statistical (fractional factorial) experimental design approach, we examined the influence of some of the characteristic parameters of virus epidemiology, social behavior, and non-pharmaceutical interventions on various measures of pandemic impact, such as total number of infected and total number of deaths in various age groups. The knowledge gained on the effects of the main factors as well as the interactions was used to develop effective NPI strategies. The efficacy of these strategies was demonstrated on simulated pandemic outbreaks triggered by influenza viruses with three different levels of severities of transmissibility. The results show that a significant mitigation of the harmful effects of a pandemic influenza outbreak can be achieved through appropriately designed NPI strategies. Our methodology, to the best of our knowledge, is the first to consider aggregated impact of NPI and social behavioral parameters and their interactions on a number of choices of performance measures for different levels of outbreak severity.

Our findings on the efficacy of NPI strategies are limited by the fact that our AB simulation model did not include mass transportation via trains, buses, and airplanes. Consequently we did not incorporate the effect of travel restrictions, an important component of NPIs, and also did not consider people arriving or leaving the outbreak region, which could influence the infection transmission process. Our contact and infection transmission models also have room for improvement. We assumed that if an infected contacts *m* individuals in a given hour, the virus shed by the infected will be shared equally by the contacts. This may not always represent the true process of virus ingestion by the susceptibles, as spatial proximity of those involved has not been considered.

Even though the profile of infectiousness of an infected is considered to vary with time and virus transmissibility, we assumed the profile to be constant among the population and independent of age and health status. Finally, we assumed that a susceptible who accumulates viral load, but does not get infected by the last hour of a day, begins the next day with zero viral load. An immunity driven dynamic model of viral accumulation in the body that considers simultaneous ingestion (from contacts) and depletion would be more realistic. However, to our knowledge, such an immune system response model has not yet been presented to the literature.

There are many assumptions in our simulation model, which directly affect the contact and infection processes, the design of the NPIs, and the NPI responses measured from the model. Through an extensive review of influenza pandemic literature, we have provided support for our assumptions. We have also made model parameter choices based on information available in the literature from past pandemic outbreaks. Statistical analysis presented in the paper allowed us to examine the impact of parameters on various performance measures. Note that, any new influenza pandemic outbreak it’s likely to bring a new virus strain and its associated epidemiological characteristics that are different from the past outbreaks. Consequently, the actual outcomes of application of NPIs may differ from the results that are presented in this paper. However, the methodology is flexible enough to adopt the changes in assumptions and parameter values to yield NPI policy guidelines for the public health decision makers.

## Electronic supplementary material

Additional file 1: **Appendix.** (PDF 1 MB)

Additional file 2: Figure S1. Number of new infections per day for baseline (no intervention) vs. NPI ^∗^ strategy obtained from the 2-level experiment for the medium transmissibility scenario. (PNG 47 KB)

Additional file 3: Figure S2. Number of new infections per day for baseline (no intervention) vs. NPI ^∗^ strategy obtained from the 2-level experiment for the high transmissibility scenario. (PNG 47 KB)
