# Supplementary material for: Design of non-pharmaceutical intervention strategies for pandemic influenza outbreaks
Source: BMC Public Health. 2014 Dec 29;14:1328. doi: 10.1186/1471-2458-14-1328 (PMC4532250; doi:10.1186/1471-2458-14-1328)
Supplement: Supplementary file 1 — Additional file 1: Appendix. (PDF 1 MB) [file 12889_2014_7561_MOESM1_ESM.pdf]

Table S1 Effects of the significant main factors and 2-level interactions from the 2^{16-7} experiment for the medium transmissibility pandemic outbreak scenario and the total number of infected as the performance measure (response)

Factors		Factor Effects	
Main Factors		Change in response when factor changes from a low to a high level	
GT		+3.07%	
DD		+3.09%	
CIT		−0.61%	
CCC		+15.42%	
CCS		+5.35%	
SCD		−5.56%	
CCDW		+2.63%	
WCD		−0.46%	
Interactions		Effect of change in Factor 1 when Factor 2 is in	
Factor 1	Factor 2	Low Level	High Level
GT	DD	+0.05%	+6.10%
GT	CCC	+5.52%	+0.63%
GT	CCS	+4.20%	+1.95%
GT	SCD	+2.49%	+3.66%
DD	HCNW	+2.76%	+3.42%
DD	CCC	+5.67%	+0.5%
DD	CCS	+4.17%	+2.00%
DD	SCD	+2.57%	+3.61%
CIT	CCS	−0.20%	−1.02%
WCD	HCW	−0.79%	−0.14%
CCC	CCS	+17.56%	+13.27%
CCS	SCD	+4.78%	+5.92%
SCD	CCDW	−7.07%	−4.05%
SCD	WCD	−5.15%	−5.97%
CCC	CCDW	+14.75%	+16.09%

Legend: GT - global threshold; DD - deployment delay; CIT - case isolation threshold; CID - case isolation duration; CICW - case isolation compliance for workers; CICNW - case isolation compliance for non-workers; HQT - household quarantine threshold.

Table S2 NPI* strategy to minimize the total number of infected for the medium transmissibility scenario (as obtained from the 2-level fractional factorial experiment based design approach)

Factor	Optimal Value	Factor	Optimal Value	Factor	Optimal Value
GT	10	DD	3	CIT	1
CID	7	CICW	0.53	CICNW	0.84
HQT	1	HQD	7	HCW	0.75
HCNW	0.84	CCC	1	CCS	1
SCD	42	CCDW	3	PDCW	0.5
WCD	14				

Legend: GT - global threshold; DD - deployment delay; CIT - case isolation threshold; CID - case isolation duration; CICW - case isolation compliance for workers; CICNW - case isolation compliance for non-workers; HQT - household quarantine threshold; HQD - household quarantine duration; HCW - household quarantine compliance for workers; HCNW - household quarantine compliance for non-workers; CCC - number of cases to close a class in a school; CCS - number of classes to close a school; SCD - school closure duration; CCDW - number of cases to close a department in a workplace; PDWC - percentage departments to close a workplace; WCD - workplace closure duration.

Table S3 Comparison of performance measures among baseline (with no intervention), ad hoc NPI strategy (NPI), and an optimal NPI strategy (NPI*) using results from the 2-level experiment for the medium transmissibility scenario

Performance Measure	Baseline	NPI	NPI*	Performance Measure	Baseline	NPI	NPI*
IAR	50.80%	36.91%	3.42%	Infections 0-19 yrs.	230,127	201,319	17,179
CFR	1.76%	1.10%	0.11%	Infections 20-64 yrs.	228,753	137,328	13,417
Pandemic Duration (Days)	93	350	84	Infections 65-99 yrs.	55,964	35,485	4,065
Total Contacts	1,047,302	709,958	67,823	Infections Households	92,217	141,603	17,977
Contacts 0-19 yrs.	520,883	484,052	40,110	Infect. MG Types(1-2)	168,185	49,784	7,588
Contacts 20-64 yrs.	416,307	181,986	20,765	Infect. Schools	247,838	178,982	8,670
Contacts 65-99 yrs.	110,112	43,920	6,948	Infect. MG Types(9-12)	6,604	3,763	426
Contacts Households	236,850	361,050	39,326	Total Deaths	17,851	11,111	1,158
Contacts MG Types(1-2)	392,793	106,101	16,587	Deaths 0-19 yrs.	1,090	975	131
Contacts Schools	403,908	235,560	11,134	Deaths 20-64 yrs.	10,681	6,332	371
Contacts MG Types(9-12)	13,751	7,247	776	Deaths 65-99 yrs.	6,080	3,804	656
Total Infections	514,844	374,132	34,661				

NPI* strategy is optimized for the total number of infected.
Legend: IAR - infection attack rate; CFR - case fatality ratio; MG - mixing group.

Table S4 Effects of the significant main factors and 2-level interactions from the 2^{16-7} experiment for the high transmissibility and the total number of infected as the performance measure (response)

Factors		Factor Effects	
**Main Factors**		**Change in response when factor changes from a low to a high level**	
GT		+2.93%	
DD		+2.83%	
CIT		−0.82%	
CCC		+17.70%	
CCS		+5.00%	
SCD		−2.76%	
CCDW		+3.87%	
PDCW		+0.73%	
WCD		−0.63%	
**Interactions**		**Effect of change in Factor 1 when Factor 2 is in**	
**Factor 1**	**Factor 2**	**Low Level**	**High Level**
GT	DD	−0.16%	+6.02%
GT	CIT	+2.68%	+3.19%
GT	CCC	+5.65%	+0.22%
GT	CCS	+4.00%	+1.86%
GT	SCD	+2.60%	+3.26%
DD	CCC	+5.67%	−0.01%
DD	CCS	+3.87%	+1.79%
CIT	CCDW	−0.43%	−1.20%
CIT	CCC	−0.51%	−1.12%
CCC	CCS	+22.75%	+12.64%
CCC	SCD	+16.66%	+18.74%

Table S4 Effects of the significant main factors and 2-level interactions from the 2^{16-7} experiment for the high transmissibility and the total number of infected as the performance measure (response) *(Continued)*

CCC	CCDW	+17.33%	+18.06%
CCC	PDCW	+17.28%	+18.11%
CCS	SCD	+4.30%	+5.69%

Legend: GT - global threshold; DD - deployment delay; CIT - case isolation threshold; CID - case isolation duration; CICW - case isolation compliance for workers; CICNW - case isolation compliance for non-workers; HQT - household quarantine threshold; HQD - household quarantine duration; HCW - household quarantine compliance for workers; HCNW - household quarantine compliance for non-workers; CCC - number of cases to close a class in a school; CCS - number of classes to close a school; SCD - school closure duration; CCDW - number of cases to close a department in a workplace; PDWC - percentage departments to close a workplace; WCD - workplace closure duration.

Table S5 Optimal NPI strategy to minimize the total number of infected for the medium transmissibility scenario (as obtained from the 2-level fractional factorial experiment based design approach)

Factor	Optimal Value	Factor	Optimal Value	Factor	Optimal Value
GT	10	DD	3	CIT	1
CID	7	CICW	0.75	CICNW	0.84
HQT	1	HQD	7	HCW	0.75
HCNW	0.57	CCC	1	CCS	1
SCD	42	CCDW	3	PDCW	0.3
WCD	14				

Legend: GT - global threshold; DD - deployment delay; CIT - case isolation threshold; CID - case isolation duration; CICW - case isolation compliance for workers; CICNW - case isolation compliance for non-workers; HQT - household quarantine threshold; HQD - household quarantine duration; HCW - household quarantine compliance for workers; HCNW - household quarantine compliance for non-workers; CCC - number of cases to close a class in a school; CCS - number of classes to close a school; SCD - school closure duration; CCDW - number of cases to close a department in a workplace; PDWC - percentage departments to close a workplace; WCD - workplace closure duration.

Table S6 Comparison of performance measures among baseline (with no intervention), ad hoc NPI strategy (NPI), and an optimal NPI strategy (NPI*) using results from the 2-level experiment for the high transmissibility scenario

Performance Measure	Baseline	NPI	NPI*	Performance Measure	Baseline	NPI	NPI*
IAR	64.53%	46.08%	16.97%	Infections 0-19 yrs.	229,952	210,941	59,476
CFR	2.55%	1.60%	0.67%	Infections 20-64 yrs.	344,381	206,455	88,524
Pandemic Duration (Days)	83	271	350	Infections 65-99 yrs.	79,718	49,630	24,017
Total Contacts	1,063,751	682,295	254,011	Infections Households	136,127	203,884	86,210
Contacts 0-19 yrs.	482,881	432,625	95,796	Infect. MG Types(1-2)	249,929	73,968	62,104
Contacts 20-64 yrs.	468,013	203,731	122,215	Infect. Schools	256,796	183,242	20,891
Contacts 65-99 yrs.	112,857	45,939	36,000	Infect. MG Types(9-12)	11,199	5,932	2,812
Contacts Households	234,411	336,832	125,456	Total Deaths	25,858	16,238	6,837
Contacts MG Types(1-2)	439,368	118,571	100,155	Deaths 0-19 yrs.	1,077	1,047	294
Contacts Schools	372,678	217,948	24,311	Deaths 20-64 yrs.	16,018	9,725	4,061
Contacts MG Types(9-12)	17,294	8,944	4,089	Deaths 65-99 yrs.	8,763	5,466	2,482
Total Infections	654,051	467,026	172,017				

NPI*strategy is optimized for the total number of infected.
Legend: IAR - infection attack rate; CFR - case fatality ratio; MG - mixing group.

Table S7 Factors and their levels for the 3-level experiment

Factor	Low	Medium	High
GT	10	30	50
DD	3	5	7
CIT	0	1	2
CCC	1	2	3
CCS	1	2	3
SCD	21	30	42
CCDW	3	4	5
WCD	7	10	14

Legend: GT - global threshold; DD - deployment delay; CIT - case isolation threshold; CCC - number of cases to close a class in a school; CCS - number of classes to close a school; SCD - school closure duration; CCDW - number of cases to close a department in a workplace; WCD - workplace closure duration.
