# Supplementary figures and images for: Design of non-pharmaceutical intervention strategies for pandemic influenza outbreaks
Source: BMC Public Health. 2014 Dec 29;14:1328. doi: 10.1186/1471-2458-14-1328 (PMC4532250; doi:10.1186/1471-2458-14-1328)

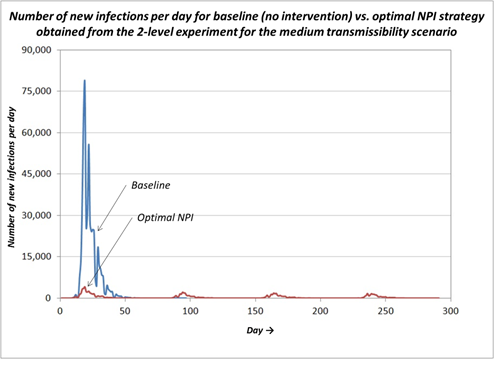

Supplement: Supplementary file 2 — Additional file 2: Figure S1. Number of new infections per day for baseline (no intervention) vs. NPI ∗ strategy obtained from the 2-level experiment for the medium transmissibility scenario. (PNG 47 KB) [file 12889_2014_7561_MOESM2_ESM.png]

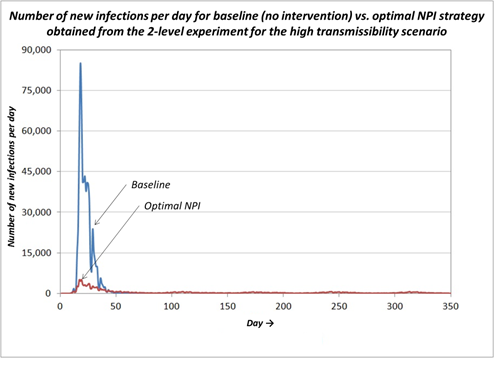

Supplement: Supplementary file 3 — Additional file 3: Figure S2. Number of new infections per day for baseline (no intervention) vs. NPI ∗ strategy obtained from the 2-level experiment for the high transmissibility scenario. (PNG 47 KB) [file 12889_2014_7561_MOESM3_ESM.png]
